# Identifying Patients' Priorities for Improving Supportive Care in Advanced Cancer: A Qualitative Study

**DOI:** 10.1111/hex.70971

**Published:** 2026-11-13

**Authors:** Sarah Scruton, Cynthia Kendell, Julee Pauling, Yelena Aizenberg, Alison Wallace, Amy Trottier, Tony Reiman, John Thoms, Robin Urquhart

**Affiliations:** ^1^ Cancer Outcomes Research Program Nova Scotia Health Halifax Nova Scotia Canada; ^2^ Department of Medicine Dalhousie University Halifax Nova Scotia Canada; ^3^ Patient Partner Ontario Canada; ^4^ Division of Thoracic Surgery, Department of Surgery Dalhousie University Halifax Nova Scotia Canada; ^5^ Division of Hematology and Hematologic Oncology, Department of Medicine Dalhousie University Halifax Nova Scotia Canada; ^6^ Department of Oncology Saint John Regional Hospital Saint John New Brunswick Canada; ^7^ School of Integrated Health University of New Brunswick Saint John New Brunswick Canada; ^8^ Discipline of Oncology, Faculty of Medicine Memorial University of Newfoundland St. John's Newfoundland Canada; ^9^ Department of Community Health and Epidemiology Dalhousie University Halifax Nova Scotia Canada

**Keywords:** advanced cancer, oncology, patient‐centred care, qualitative, supportive care

## Abstract

**Background:**

Developments in precision oncology have introduced a new population of patients living long‐term while receiving on‐going treatment for advanced cancer. These patients experience unique needs and challenges that are not adequately addressed by existing survivorship resources or current models of care. To address these challenges and better support this population, this study aimed to establish patient‐identified priority areas for intervention development and health systems change.

**Methods:**

Individuals across Canada receiving on‐going treatment for advanced cancer took part in semi‐structured interviews (*n* = 22). They were asked to describe the supports they would like to access to improve their quality of life and address the challenges they face. Qualitative descriptive methods were used to analyse the transcribed interviews, in close collaboration with patient partners.

**Results:**

Participants described priority areas encompassing healthcare system improvements, information provision and navigation, mental health and emotional support, healthy living, and practical support. They highlighted the need for integrative communication and collaboration between healthcare providers, as well as guidance towards existing resources and supports. The development or adaptation of supports that are tailored to the unique needs these patients face, and that are offered by providers who understand their needs and experiences, is essential.

**Conclusion:**

To meet the needs of this new population of individuals with advanced cancer, greater investment is needed to tailor existing supports to their specific needs and to strength health system infrastructure to enable collaboration and coordination amongst providers. These findings contribute to ongoing advocacy for prioritising cancer research funding to improve care for this growing population.

**Lived Experience or Public Contribution:**

Patient partners were actively engaged as members of the research team throughout all stages of the study, including concept development, creation of study materials, data analysis, and manuscript preparation and review. In addition, several members of an existing patient working group (Marathon of Hope Cancer Centres Network Patient Working Group) contributed to specific elements of the study design and procedures.

## 1 Introduction

Cancer is a leading cause of death in Canada and worldwide, with an estimated 698 Canadians diagnosed and 239 dying from the disease each day in 2025 [1]. Worldwide, cancer accounted for nearly 10 million deaths in 2024 [2]. Survival likelihood depends greatly on the stage at diagnosis, as around 90% of those diagnosed at stage I are expected to survive past 5 years, compared to 10‐20% of those diagnosed at stage IV [3]. The era of precision oncology is quickly shifting these statistics, however, as cancer treatments are developed based on a person's genetic makeup and the genetic and molecular makeup of the cancer itself [4]. Now, targeted therapies can inhibit specific oncogenic pathways or driver alterations within cancer cells, while immunotherapies enhance the immune system's ability to recognise and eliminate cancer cells [5, 6]. This has resulted in truly unprecedented improvements in survival for certain cancer sub‐types. For example, in 2016, 5‐year survival for metastatic non‐small cell lung cancer was less than 6% [7]. Now, 5‐year survival exceeds 60% for certain sub‐types (ex. ALK+) treated with targeted therapies [8]. A similar pattern is observed in multiple myeloma, where 5‐year survival has doubled from 31% in 2001 to > 60% in 2022 [9, 10].

These developments have led to a growing population of patients living long‐term with advanced cancer while receiving on‐going treatment. While this progress is encouraging, it introduces challenges for a health system not designed for the long‐term management of these patients. Typically, cancer care follows two pathways: patients treated for early‐stage cancer who enter the post‐treatment phase, and patients treated for late‐stage cancer who eventually enter the end‐of‐life phase [11]. Arguably, this new population fits neither pathway. Existing survivorship care research and resources have largely focused on post‐treatment populations and may therefore not be appropriate for this emerging late‐stage population [12, 13]. As a part of a larger study, we previously identified several challenges faced by individuals living long‐term with advanced cancer. Participants struggled with persistent symptoms and side effects, changes in daily routines, new identities, and feelings of stigma and isolation [14]. Participants also faced barriers to accessing supports and noted that resources were not designed with their needs in mind [14]. Existing interventions are often short‐term and may not address on‐going needs (ex. fatigue, cognitive difficulties, anxiety), and they tend to focus on challenges related to either post‐treatment reintegration or preparing for end‐of‐life [15, 16]. In addition, barriers within the healthcare system, such as poor provider coordination, insufficient time to discuss patient concerns, and inadequate funding for ongoing supports, leave individuals to navigate these challenges alone [17, 18, 19].

In recent years, organisations such as the Marathon of Hope Cancer Centres Network (MOHCCN) and the Multinational Association of Supportive Care in Cancer (MASCC) have called for improved survivorship care for people with advanced cancer [12, 20]. However, the development of new care models and related interventions must include the lived experiences of patients to ensure that services and supports address their needs, preferences, and circumstances. Previous research has identified personal mechanisms used by patients with advance cancer to cope with their diagnosis (ex. maintaining routine, evaluating priorities, setting personal goals) [21], but patient‐identified health system and supportive care priorities remain poorly understood. As such, the objective of this study was to identify priority areas for intervention development and health system improvement based on the lived experiences of patients receiving on‐going treatment for advanced cancer. The goals of patient partner involvement in this study were to refine the research aims and questions, facilitate interviews to address the objectives of the study, and interpret the findings through the lens of lived experience.

## 2 Methods

### 2.1 Study Development

This manuscript reports on one component of a larger qualitative study exploring the lived experiences of individuals diagnosed with advanced cancer [14]. The broader study examined challenges faced by individuals receiving ongoing treatment, barriers and facilitators to addressing their challenges, and considerations for intervention development, with this manuscript focusing on the latter. This study was approved by the Nova Scotia Health Research Ethics Board on November 28th, 2024 (Protocol #1031201).

### 2.2 Patient and Public Involvement

Three patient partners were actively and meaningfully engaged as a part of the research team. Their involvement across all stages of the study is detailed throughout the Methods section. These individuals had received an advanced cancer diagnosis and could apply their lived experience to the design and interpretation of the study. The patient partners included two working‐age women living in urban settings, and one retired man living in a rural setting.

In addition, this study was designed to address the research goals established by the MOHCCN Patient Working Group (PWG) as a part of the Patient Voices in Research Initiative [20, 22]. The PWG is a group of over 30 volunteers with lived cancer experience who work closely with researchers and physicians to shape cancer care in Canada. The group represents diverse ages, genders, ethnicities, educational backgrounds, cancer sites, and geographic regions across Canada. The PWG helped to guide the study development and interpretation of findings through regular updates and meetings with the research team.

Patient and public involvement in this study is reported using the Guidance for Reporting Involvement of Patients and the Public (GRIPP2) shortform checklist (Appendix 1) [23].

### 2.3 Participants and Recruitment

We aimed to recruit 20‐25 participants across Canada who were actively receiving ongoing treatment for an advanced cancer diagnosis (i.e., cancer that has spread to other areas of the body or is otherwise incurable). Eligible participants were above the age of 18, English speaking, and had lung, prostate, ovarian, multiple myeloma, melanoma, or breast cancer. Purposive sampling was used for variation in cancer type, age, gender, and geography [24]. Recruitment posters were distributed through cancer organisations and social media, and through personal networks of the research team and the MOHCCN PWG. Interested participants contacted the Research Coordinator (RC) who confirmed their eligibility, provided information about the study and research team (i.e., background information and objectives), consented them into the study, and scheduled their interview. Due to the focus of the research, five participants were aware of, or had limited prior interactions with, the research team prior to participation.

### 2.4 Data Collection

Participants attended a 1‐2 h one‐on‐one semi‐structured interview conducted by the RC who is female, Master's trained, and experienced in qualitative methods. Interviews occurred virtually via Zoom for Healthcare or Microsoft Teams. No drop‐outs or refusals to participate occurred after the RC contacted potential participants to explain the study and conduct the consent process. However, four individuals who initially contacted the RC by email did not respond to subsequent follow‐up communication. Demographic information for these non‐respondents was not available. The interviews were 1‐2 h in length, and were audio and video recorded and transcribed verbatim by a professional transcriptionist to aid in the analysis, along with field notes recorded by the RC to identify emerging themes. Participants did not review the transcripts or findings for accuracy or to provide additional comments. The interview guide was developed in collaboration with the patient partners to capture participants' ideas for health system change and intervention development to better support individuals living with advanced cancer. A draft interview guide was first developed by the RC and PI based on the study objectives and a smaller pilot study done by the research team [25] (Appendix 2). The patient partners then revised the interview guide to enhance its relevance, comprehensiveness, and clarity, ensuring it would elicit in‐depth and meaningful responses from patients regarding their experiences and perspectives on supportive care interventions. No repeat interviews were conducted. Data collection began in February 2025 and ended in August 2025 when theoretical saturation was achieved, meaning no new insights emerged from the data that were relevant to the research question [26].

### 2.5 Analysis

Qualitative descriptive methods guided the analysis, which aim to clearly and comprehensively describe the participant's experience as is, rather than make interpretations with theorisation or abstraction, where possible [27, 28]. The RC and patient partners first read and reviewed the de‐identified transcripts before uploading them into NVivo 15. During this initial review, the patient partners took preliminary notes to identify and describe emerging themes and concepts, which informed the development of a coding framework (Appendix 3) in collaboration with other members of the research team. The data were then organised line by line into broad conceptual categories using deductive coding based on this framework. As the analysis progressed, additional codes were added inductively to capture new constructs that emerged from the transcripts. Once complete, the codes were reviewed by the RC and the patient partners and supervised by the PI to identify recurring and distinctive themes that reflected participant experiences and provided insight into the research question. The RC maintained an audit trail to document key analytic decisions such as changes to the coding framework and the development of themes. Peer debriefing of the findings was carried out with clinicians and researchers involved in advanced cancer care, along with members of the MOHCCN PWG. Reflexivity was considered throughout the study, recognising that the research team's diverse professional and personal experiences with advanced cancer may shape their perspectives and influence data interpretation. This study adheres to the consolidated criteria for reporting qualitative research (COREQ; Appendix 4) [29].

## 3 Results

### 3.1 Results of Patient and Public Involvement

Patient partners contributed to the development and refinement of the interview guide and participated in the analysis of the data, as described in the Methods. Their perspectives informed the interpretation of participants' accounts and contributed to the development of the final themes and findings reported below. No negative outcomes or challenges arising from patient and public involvement were identified.

### 3.2 Participants

In total, twenty‐two individuals from across Canada took part in the interviews. On average, participants were 60.4 years old and had received their diagnosis 5.8 years prior. The most common cancer type was breast (32%) followed by multiple myeloma (27%). The participants mostly identified as women (73%), had received a post‐secondary education (86%), and had access to a primary care provider (91%). The participants mostly resided in Ontario (36%), in an urban area (83%), and within 30 min of travel to cancer care (64%). Detailed demographic characteristics are shown in Table 1.

**Table 1:** Detailed demographic characteristics.

Demographic characteristic	*N* (%)
Age	
Mean, range (years)	60.4, 40–81
Province	
Ontario	8 (36.4)
Alberta	7 (31.8)
British Columbia	4 (18.2)
Nova Scotia	2 (9.1)
Manitoba	1 (4.5)
Gender identity	
Woman	16 (72.7)
Man	6 (27.3)
Rural or urban residence	
Urban	18 (81.8)
Rural	4 (18.2)
Level of education attained	
High school	2 (9.1)
Post‐secondary	19 (86.4)
Missing	1 (4.5)
Cancer Type	
Breast	7 (31.8)
Multiple Myeloma	6 (27.3)
Melanoma	4 (18.2)
Lung	2 (9.1)
Ovarian	2 (9.1)
Prostate	1 (4.5)
Years since diagnosis	
Mean, range	5.8, 1–12
Distance to cancer care	
< 30 min	14 (63.6)
30 min ‐ 1 h	6 (27.3)
1+ hours	2 (9.1)
Access to a family physician	
Yes	20 (90.9)
No	2 (9.1)

### 3.3 Improving Supportive Care for Individuals Living With Advanced Cancer

When asked the types of support they wished to access, or they believed would improve their quality of life, participants described both healthcare system improvements and the development or adaptation of supportive care resources. The latter encompassed information provision and navigation, mental health and emotional support, healthy living, and practical support. Participants also discussed considerations for designing interventions that are appropriate and accessible to all.

#### 3.3.1 Healthcare System Improvements

Twelve participants reflected on changes within the healthcare system that could improve their care experience. These largely centred on the need for multidisciplinary collaboration, particularly related to better integration between oncology and supportive care services and providers (i.e., mental health practitioners, dieticians, etc.).

But ideally, to me, [cancer care] is a multidisciplined approach when it comes to stage 4 cancer. Meaning that you have a multitude of individuals to seek and get resources from.

(P1, man, melanoma)

I think [best possible care] would be a fast response when [a need] occurs. And kind of wraparound services for the individual's needs ‐ whatever they may be…. Well, say somebody's concerned about their diet and cancer, to have those services available.

(P8, woman, breast cancer)

Participants emphasised the importance of more efficient and accessible communication with providers, including the use of virtual appointments and other digital technologies. They also highlighted the need for improved access to medications and awareness of clinical trials, as well as opportunities to engage in advocacy efforts.

How do we make sure that [patients] have the opportunity, when it's opened up, to participate in studies and/or trials? Especially trials because the trials are the ones that actually can impact us now. So how do we do that? And there's no answer that I can think of, or suggestion. Actually, no, I do have one. We have a nurse navigator? Why is there not a study or research navigator in each cancer centre who makes sure that the oncologists know what's available?

(P12, woman, breast cancer)

Like I would love to do more of [patient advocacy]. So, if there's anything that you're aware of that I can be connected to, that would be great. But, yeah, I think sometimes [patient advocate] roles exist. And they're kind of in name only, and to just sort of say you have one. So, I don't know whether they could potentially be leveraged.

(P13, woman, breast cancer)

#### 3.3.2 Information Provision and Navigation Support

The most frequently described priority area (discussed by nineteen participants) was the need for more information. Participants emphasised the importance of trusted, credible, and up‐to‐date information about their cancer, side effects, and treatment. They noted that this information needs to be readily available to patients, rather than requiring patients to search for it themselves.

When you're first diagnosed, it would be helpful to have written information so you can look at it. Giving you reputable information about, you know, what sites to go on (…) What your treatment will look like, what it entails, maybe the side effects.

(P18, woman, myeloma)

There's just no conversation around like, Well, wait, why did I have [cancer] in the first place? And like isn't this the things we should be changing about my life that like caused me to get cancer in the first place? And there's no conversation about why is this the treatment that's being recommended? And are you sure it's actually going to work? And what are the side effects? And like there's just… I have a thousand answers to the question of where would I want better information.

(P13, woman, breast cancer)

I don't drive for 30 min there and back, just for a 5 min conversation in his office. I just thought there'd be lots more to discuss, you know, outside of two or three questions that I might have that I usually don't get a full answer from, you know. So open communication, for sure, you know, would help.

(P16, man, myeloma)

Relatedly, five participants suggested the creation of a centralised resource directory to help patients identify local or remote supports to address their needs. They envisioned, for example, an accessible online hub that directs users towards financial, psychosocial, practical, and disease‐specific resources that could be accessed independently at any time.

I think like having a better library of resources for people, as opposed to just what's available at your hospital. (…) I think there could be a greater, yeah, repository of resources that people can access where and when they need it. And it's everything from, you know, financial supports, psychosocial supports, here's information on your disease, and here's, you know, where you can go to find out more about it.

(P10, man, lung cancer)

If we could ever get [a portal] where… And maybe AI is the way, because I've started using AI, of getting information about what to do, where to go – all those types of components. And stop all this trial and error, hunt and peck, type of thing, trying to get some resources that are available for you to use. And that can be anything from what clinical trials are out there, to nutrition, to the value of exercise, to what's available within your community.

(P22, man, prostate cancer)

Twelve participants discussed the need for person‐to‐person navigation support to guide them through a complex healthcare system. This included assistance with accessing treatments or medications, obtaining necessary information, and identifying and coordinating access to existing supports and services. The participants suggested that navigation could be provided by either a healthcare provider (e.g., a nurse or social worker) or by a peer with lived experience of cancer.

Maybe someone to sit you down and say, ‘Okay, you have this type of cancer. Here's the supports we have in the community for this kind of cancer, here's the websites.’ You know, I think that would help. Because at no point has anybody at [Hospital] told me about a support group in person. I've had to look it up myself. And that's fine. I'm obviously capable, you know. But it'd be kind of cool if someone said, ‘Hey, we see you're struggling. Why don't you maybe talk to somebody?’ Or, you know, heck, even my hematologist could say, ‘Look, I've got two patients that would really help you that are on the other side of this. Maybe you should talk to them.’ You know, put me in touch with another patient that's 2 years advanced past the stage that I'm at.

(P16, man, myeloma)

For the newly diagnosed, I think [navigation support] would help them navigate what they're going through, and where they can find things. You know, if they have one person that can, you know, show them everything, or give them advice on where to go for things and what they're looking for, then that would be good.

(P18, woman, myeloma)

#### 3.3.3 Mental Health and Emotional Support

Fourteen participants discussed the need for improved, ongoing, cancer‐specific mental health and emotional support. Participants wanted greater access to mental health services provided in a timely manner and delivered by professionals who understand the unique experiences and needs of those living with advanced cancer.

Yeah, [psychologist] called me once. He said, ‘I'm going to be your kind of guy that…your guy that you talk to.’ He called me once, and I haven't heard back from him at all. So that's something I kind of wish that [psychologist] would have actually kept in touch. I didn't even hear back a second time from him. So I mean, you know, had he called me back and said, ‘Hey, how you doing? I haven't talked to you for a month, or whatever, 2 months.’ But there was no contact after that. That's where… That's one thing that could be improved. Because when you hear that, that word cancer, you just think that it's over, right. I mean this is it, the end is near.

(P3, man, melanoma)

I seen a therapist at [Hospital]. They have a therapist that you could talk to. But, you know, I think when it comes to a therapist, they don't… If they don't have cancer, it's hard for them to relate. It really is. I mean a therapist that's been through it, that'd be cool.

(P16, man, myeloma)

Five participants emphasised that this support could be extended to family members to help them cope with their loved one's diagnosis, for example, through counselling for spouses or children.

In the same way that stage 4 for a stable patient is different for patients, it's also different for caregivers. (…) But it's that same idea of like my partner has been through this rollercoaster of… The same rollercoaster as I have.

(P13, woman, breast cancer)

Well, if I was a parent and had a kid, I'd want to have guidance on how…depending on the child's developmental stage, how to express [advanced cancer] to the child in the most healthy way.

(P8, woman, breast cancer)

Participants also suggested expanded opportunities for peer support and emotional expression. They discussed the importance of being able to connect with others who share similar experiences, in spaces where they feel safe and welcomed.

If there's something that would make a real difference to me, it would be being actually able to meet with other people who are dealing with cancer. It doesn't have to be my same kind, but where people can really talk honestly with each other. And I think there's something about that type of support that you can't easily replace.

(P7, woman, breast cancer)

Maybe just, again, you know, to meet with other myeloma patients. Because I think we're unique in what we can offer one another. And, you know, we meet for the first time, and we ask one another the most personal questions, you know, that would be considered rude if you ask somebody else.

(P20, woman, myeloma)

#### 3.3.4 Healthy Living

Seven participants expressed wanting guidance and resources to help maintain or regain physical functioning and to support a health‐conscious lifestyle. This highlighted a need for healthy living supports, including individualised nutrition guidance and exercise programmes that are tailored to each patient's goals and needs. For example, while nutrition support for advanced cancer tends to focus on weight gain or maintenance, one participant expressed difficulty finding support for weight loss:

For example, I would like to lose weight. And I don't think [healthcare providers] will want to talk to me about that. I have read about what [healthcare providers] do. And they will try to help those patients who don't have appetite at all and are losing weight like just because their chemotherapies have been affecting their appetite, or some who have some radiation to the mouth or whatever, and they need to do a liquid diet. And, yeah, that is the situation that they address. But not really healthier eating or something like that.

(P11, woman, breast cancer)

And the other thing I want to talk…we need to talk about is dietary concerns. And I think that they need to open the door to people who are taking care of diets, but not necessarily in the cancer realm. We need people who really know diet also involved. It's not saying that our dietitians don't have a good grasp on everything, but we need other people with other ideas that you can learn about.

(P4, man, melanoma)

Physical activity was another area wherein participants expressed a desire for support. They emphasised that physical activity resources must be individualised to patients' abilities, which are likely to change over time, to ensure they are safe and effective.

Like my physio, the gentleman I seen about my back, he like gave me some things and like, ‘Go home and do it.’ But, you know, they don't understand you're weak, too. Like, you know, you're constantly fighting fatigue. And it's not as simple as, you know, start with 10 of these, and in a couple days go to 15 or 20. And you're just… It wipes you out, you know? So I think that's maybe the kind of knowledge, you know, that would be good, too. You know, it's one thing to go to a physiotherapist and they hear you have cancer. But really, until… It's almost the case if you haven't had it yourself, it's really hard to relate.

(P16, man, myeloma)

I think, you know, physical activity, whether it be in a chair or restorative yoga, something that's not necessarily requiring too much energy or movement, would be good to have as well.

(P8, woman, breast cancer)

#### 3.3.5 Practical Support

Finally, four participants described the need for in‐home and logistical supports such as transportation, housekeeping, and grocery delivery to help manage the day‐to‐day challenges associated with ongoing treatment.

Those little mundane tasks that build up. And it's like you sometimes will get overwhelmed. If you're having a bad day, you're not feeling up to it, and it's like, Okay, I've got this big pile of laundry, I've got food, I have to get groceries, I've got this, this and this. So, if there was a service that could take one of those things off your plate.

(P9, woman, breast cancer)

If there was someone to help me like to prepare a meal for my kids, or someone to pick up the groceries, that would be super useful.

(P12, woman, breast cancer)

Five participants also discussed the importance of financial supports to help navigate income loss and out‐of‐pocket expenses. This included assistance with taxes and disability benefits, as well as strategies to alleviate financial burden (i.e., free parking passes during appointments).

Income tax. You know, like figuring out, am I, you know, disabled under Revenue Canada, or am I…. Because I'm on a disability thing, and what can I deduct? And we did have our taxes done. And in order to get the CPP Disability, they had to… I had to have an accountant hired. But they don't have anyone with the clinic that knows about sick people, like cancer patients, that would be able to advise.

(P14, woman, breast cancer)

I was very fortunate in my ability to go off on Disability. But even then, I was only… You know, we're only bringing in half of what I would normally make. And if I'm the sole caregiver, then we are in trouble. And that is the last thing someone with cancer needs to worry about, is how they're going to feed their family, and how their family is going to be taken care of. And the Canadian government needs to look at that, and provide more financial support for people dealing with cancer, a hundred percent.

(P10, man, lung cancer)

#### 3.3.6 Design Considerations

Thirteen participants raised several important considerations for the design and implementation of future interventions. It was evident that existing supportive care resources are not designed in a way that optimally addresses their needs, and a “one‐size‐fits‐all” approach may not be effective. Rather, supports and resources need to be co‐designed with patients and, to the extent possible, tailored to individual circumstances such as location, accessibility requirements, age, family dynamics, and coping styles.

Just understand that everyone's journey is different. You can't make one blanket program, build one blanket program for all. You really have to look at, you know, people's, you know, locations, like where they live and their communities.

(P10, man, lung cancer)

So, I guess the perspective I'd offer you on that in terms of how to design [future supports] would be with input from the prospective users of the group around those practical issues. Can you make it in person? Do you have a time of day that works or doesn't work for you? Do you have someone to drive you?

(P7, woman, breast cancer)

They also emphasised the importance of appropriate timing and delivery of interventions, ensuring they are introduced early enough to be beneficial, but not at a time when patients are already overwhelmed with information.

The other thing we talked about is the timing of the delivery of [supports]. The studies, I was happy to do it day one. But there were things like here are the support groups that like you can access and stuff like that. I think that if [healthcare providers] delivered that separately, I would have paid attention to it. But right at that moment, like my first intake and my first appointment, all I cared about was the physical.

(P12, woman, breast cancer)

Lastly, some participants noted that supports may already exist but are underutilized, highlighting the need for implementation strategies that prioritise awareness and equitable access.

## 4 Discussion

Existing supportive care interventions are not designed for those living long‐term with advanced cancer, and the participants in this study identified several priority areas for health system re‐design and resource development to better address their needs. Within the health system, they called for improved collaboration and communication between clinicians, as well as need for multidisciplinary care. The most common priority discussed was the need for more cancer‐related information and direction toward existing resources, as well as support navigating the health system. Participants also called for tailored mental health support for both themselves and their loved ones, provided by professionals who are trained to manage their specific needs. Participants believed their physical health could be addressed by nutrition support or exercise programmes designed for their specific needs, goals, and abilities, rather than those of the general cancer population. Finally, they suggested in‐home and practical supports to help manage day‐to‐day living.

Several of these priority areas require adaptation of and navigation towards existing supports and healthcare resources, rather than investing solely in the development of new interventions. Firstly, the need for information provision and navigation support has been addressed by various initiatives across Canada designed to reduce feelings of overwhelm, minimise the risk of misinformation, and reduce barriers to care. Examples of informational resources include the Canadian Cancer Society's Advanced Cancer booklet [30] and the I‐Can Manage self‐management programme [31]. Several navigation programmes have also been implemented resulting in improved care coordination, quality of life, patient activation, and level of support. These include both provincial professional‐led models [32], and volunteer‐led programmes such as PeerNav, a web‐based, peer‐led navigation programme for men with prostate cancer [33], or Nav‐CARE, a navigation programme for older persons with chronic illness [34]. That said, there is currently no published research on navigation, self‐management, or resource directory programmes that have been successfully implemented specifically for individuals with advanced cancer [35]. This highlights a priority to adapt such programmes to address the specific needs experienced by this group as they develop and change throughout the cancer trajectory.

In addition, where supportive care resources are available across Canada, access to and utilisation of these services remain limited among patients. In the present study, participants expressed a need for greater opportunities to connect with peers and individuals with shared experiences. Several provincial and national peer‐support programmes are available across Canada, including both in‐person and virtual models. For instance, the Canadian Cancer Society's CancerConnection.ca provides a platform for individuals with cancer to connect with peers online [36]. Similarly, Wellspring offers one‐to‐one peer support facilitated by trained volunteers with lived experience of cancer [37]. Other examples include BC Cancer's Living with Metastatic Cancer virtual support group, and Myeloma Canada's virtual Facebook support groups [38, 39]. These types of supports are important, as their positive effects on psychological distress, resilience, loneliness, quality of life, and coping are well documented in the literature [40, 41]. However, many patients face barriers accessing these supports, including difficulties researching relevant resources themselves and receiving limited information about available supports from the healthcare system [14, 42, 43, 44]. As such, efforts to improve access to supportive care through tailored navigation supports and interventions that increase awareness of existing programmes are equally important to the development and adaptation of supportive care resources.

Palliative care is an additional existing resource that is likely to address many of the needs experienced by this population. However, access remains limited, despite ASCO guidelines recommending early integration of palliative care alongside cancer treatment to improve symptom management and quality of life [45]. In addition to patient and provider perceptions that associate palliative care with end‐of‐life care [46], limited resources and service capacity further constrain access and availability. As such, some Canadian programmes will not accept palliative care referrals unless patients are no longer receiving treatment and have an estimated prognosis of less than 6 months to 1 year [47, 48, 49]. In response to these barriers, efforts to expand and promote earlier integration of palliative care are underway in Canada. These include the development of competency frameworks to build capacity by equipping a broader range of healthcare providers with the knowledge and skills required to deliver palliative care [50, 51, 52]. This also includes increasing patient awareness and referrals, improving provider communication with patients about palliative care, and distinguishing palliative care from hospice or care focused exclusively on the end of life. Thus, strengthening the integration of palliative care represents an additional opportunity to increase the capacity of existing health system resources, rather than relying solely on the development of new strategies or services.

A further limitation of existing supportive care resources in Canada is that they are often limited in duration and not tailored to the individual needs of patients receiving ongoing treatment for advanced cancer. For example, exercise programmes typically run for a set number of weeks [53], survivorship programmes tend to focus on post‐treatment reintegration [15], and emotional supports for advanced cancer often target end‐of‐life care [54, 55]. A key barrier to adapting these programmes may be limited provider knowledge of how to care for this population. For example, rehabilitation providers have reported limited information and training specific to the management of advanced cancer patients [56, 57], and mental health practitioners have reported difficulty treating this population due to insufficient training in both cancer‐specific knowledge and the psychosocial impacts associated with advanced cancer [58]. As such, addressing this gap may require targeted provider training and additional funding and infrastructure to implement programmes as a part of ongoing care. This represents a significant challenge in Canada, given that only 4% of cancer research funding is invested in survivorship research [59]. Therefore, it is important to demonstrate how greater investments could not only improve patient outcomes but also reduce healthcare costs by addressing issues early and preventing escalation that requires intensive health service use.

The priority areas requiring healthcare system re‐design will likely require substantial resource investment and restructuring to address. This new population of individuals receiving ongoing treatment are reliant on a healthcare system that is already constrained by patients who have completed primary treatment but have not yet been transitioned back to primary care [60]. A population‐based study from Nova Scotia, Canada, found that cancer centre follow‐up visits (for people post‐treatment with no evidence of disease) increased at a rate of 8% per year from 2006 to 2015, while the rate of increase for all cancer centres visits in the same time period was 14% per year [60]. One of the principal issues, therefore, is that Canada does not have enough cancer specialists to meet this growing demand [61]. A potential solution, identified by researchers, policy makers, and cancer organisations [62, 63, 64] in addition to the participants in this study, is improved integration of multidisciplinary care. This is a collaborative approach in which oncologists, nurses, palliative medicine specialists, allied healthcare providers, and more, are integrated into a patient's care to address psychosocial, physical, and practical needs in addition to cancer management [62]. Ultimately, this approach would reduce burden on individual providers by distributing it amongst several team members and aligning patient needs with provider expertise [64, 65, 66, 67]. Upfront, however, substantial investment in human resources is required to have sufficient specialty‐trained providers and to establish the training and infrastructure necessary for collaboration and communication [62, 68, 69, 70]. This priority falls within the Canadian Cancer Society's call for targeted funding towards a robust nationwide health human resources strategy, to increase the number of adequately trained, equally distributed and performing healthcare providers [71]. The findings of this study echo this call, highlighting the need for the health system to consider developing innovative strategies (i.e., expanding roles of allied healthcare professionals) and expanding the capacity and reach of existing resources (i.e., palliative care) to meet the needs of the growing population of individuals living long‐term with advanced cancer. The results of this study highlight key patient‐identified areas of unmet needs that can inform these strategies. However, advancing change within these priority areas will also require input from additional relevant groups, including family members, health system researchers, decision‐makers, and healthcare providers. Engaging these groups represents an important next step to understanding and addressing barriers and facilitators to increasing access to, developing, and adapting supports and resources for individuals with advanced cancer.

The involvement of patient partners and the MOHCCN PWG influenced the study by bringing patient perspectives into the design of the study and interpretation of the findings. Their involvement provided an additional perspective alongside those of researchers and clinicians to ensure the interpretation of participants' experiences remained grounded in patient perspectives. We did not identify any negative effects of PPI.

## 5 Limitations

This study is not without its limitations. The self‐selected sample had limited representation of men, individuals without post‐secondary education, and individuals living in rural or remote areas. As such, the results cannot be generalised to all individuals who are diagnosed with advanced cancer and may not reflect the experiences of those who face additional barriers to care. However, we achieved theoretical saturation with a relatively large sample size, which is a key strength that supports the credibility and robustness of the findings and allows us to demonstrate patient priorities for improving cancer care. In addition, race and ethnicity were not collected, limiting our ability to assess how these factors may have shaped participants' experiences and priorities.

## 6 Conclusion

Individuals receiving ongoing treatment for advanced cancer experience challenges that are not addressed by existing resources and supports. This study identified several priority areas for future research and intervention development, including improved collaboration and communication between providers, informational and navigation support, tailored exercise, nutrition, and mental health support, and practical assistance for day‐to‐day living. Future initiatives must address these individuals' unique, evolving needs and be available throughout the entire cancer trajectory.

## Author Contributions

**Sarah Scruton:** conceptualisation, methodology, data curation, investigation, formal analysis, project administration, writing – original draft, writing – review and editing. **Cynthia Kendell:** conceptualisation, formal analysis, writing – review and editing, methodology. **Julee Pauling:** conceptualisation, methodology, formal analysis, writing – review and editing. **Yelena Aizenberg:** conceptualisation, methodology, formal analysis, writing – review and editing. **Alison Wallace:** conceptualisation, writing – review and editing, methodology. **Amy Trottier:** conceptualisation, methodology, writing – review and editing. **Tony Reiman:** conceptualisation, methodology, writing – review and editing. **John Thoms:** conceptualisation, methodology, writing – review and editing. **Robin Urquhart:** conceptualisation, methodology, investigation, formal analysis, supervision, funding acquisition, project administration, writing – review and editing.

## Ethics Statement

Ethical approval for this study was granted by the Research Ethics Board of Nova Scotia Health on November 28, 2024 (protocol number 1031201).

## Conflicts of Interest

The authors declare no conflicts of interest.

## Supporting information


Supporting File 1

Supporting File 2

Supporting File 3

Supporting File 4

## Data Availability

The data that support the findings of this study are available from the corresponding author upon reasonable request. The data are not publicly available due to privacy or ethical restrictions.

## References

[1] Canadian Cancer Society . Cancer Statistics at a Glance. 2026. https://cancer.ca/en/research/cancer-statistics/cancer-statistics-at-a-glance.

[2] World Health Organization . Cancer. 2026. https://www.who.int/news-room/fact-sheets/detail/cancer.

[3] Government of Canada . Five‐year Cancer Survival by Stage at Diagnosis in Canada. 2023. https://www150.statcan.gc.ca/n1/pub/82-003-x/2023001/article/00001-eng.htm. 10.25318/82-003-x202300100001-eng 36716075

[4] Canadian Cancer Society . Precision Medicine. 2021. https://cancer.ca/en/research/understanding-cancer-research/precision-medicine.

[5] Canadian Cancer Society . Targeted Therapy. 2025. https://cancer.ca/en/treatments/treatment-types/targeted-therapy.

[6] Canadian Cancer Society . Immunotherapy. 2025. https://cancer.ca/en/treatments/treatment-types/immunotherapy.

[7] P. Goldstraw , K. Chansky , J. Crowley , et al., “The Iaslc Lung Cancer Staging Project: Proposals for Revision of the Tnm Stage Groupings in the Forthcoming (Eighth) Edition of the Tnm Classification for Lung Cancer,” Journal of Thoracic Oncology 11, no. 1 (January 2016): 39–51, 10.1016/j.jtho.2015.09.009. 26762738

[8] B. J. Solomon , G. Liu , E. Felip , et al., “Lorlatinib Versus Crizotinib in Patients With Advanced Alk‐Positive Non–Small Cell Lung Cancer: 5‐Year Outcomes From the Phase Iii Crown Study,” Journal of Clinical Oncology 42, no. 29 (October 2024): 3400–3409, 10.1200/JCO.24.00581. PMC11458101 38819031

[9] SEER . Myeloma ‐ Cancer Stat Facts. 2026. https://seer.cancer.gov/statfacts/html/mulmy.html.

[10] S. K. Kumar , A. Dispenzieri , M. Q. Lacy , et al., “Continued Improvement in Survival in Multiple Myeloma: Changes in Early Mortality and Outcomes in Older Patients,” Leukemia 28, no. 5 (May 2014): 1122–1128, 10.1038/leu.2013.313. PMC4000285 24157580

[11] Z. Fekete , A. Fekete , and G. Kacsó , “Treatment Classification by Intent in Oncology—The Need for Meaningful Definitions: Curative, Palliative and Potentially Life‐Prolonging,” Journal of Personalized Medicine 14, no. 9 (August 2024): 932, 10.3390/jpm14090932. PMC11433215 39338185

[12] N. H. Hart , L. Nekhlyudov , T. J. Smith , et al., “Survivorship Care for People Affected by Advanced or Metastatic Cancer: Mascc‐Asco Standards and Practice Recommendations,” Supportive care in cancer: official journal of the Multinational Association of Supportive Care in Cancer 32, no. 5 (April 2024): 313, 10.1007/s00520-024-08465-8. PMC11056340 38679639

[13] A. L. Smith , N. H. Hart , M. Jefford , and R. J. Chan , “Survivorship Research for People With Metastatic or Advanced Cancer: A Time for Action,” Asia‐Pacific Journal of Oncology Nursing 9, no. 4 (February 2022): 185–186, 10.1016/j.apjon.2022.02.001. PMC9096729 35571628

[14] S. Scruton , C. Kendell , J. Pauling , et al., “Living With and Beyond Advanced Cancer: Understanding Patient Needs and Experiences,” BMC Cancer, ahead of print, September 4, 2026, 10.1186/s12885-026-16808-3.

[15] S. Murnaghan , S. Scruton , and R. Urquhart , “Psychosocial Interventions That Target Adult Cancer Survivors' Reintegration into Daily Life After Active Cancer Treatment: A Scoping Review,” JBI Evid Synth 22, no. 4 (April 2024): 607–656, 10.11124/JBIES-23-00044. PMC10986786 38015073

[16] N. M. Chew , E. L. Ting , L. Kerr , D. J. Brewster , and P. L. Russo , “Psychosocial Interventions at the End‐Of‐Life: A Scoping Review,” Cancer Nursing 46, no. 6 (December 2023): 432–446, 10.1097/NCC.0000000000001136. 35786585

[17] A. Hult , E. Lundgren , C. Fröjd , A. Lindam , and E. Jangland , “Patient Complaints about Communication in Cancer Care Settings: Hidden Between the Lines,” Patient Education and Counseling 114 (September 2023): 107838, 10.1016/j.pec.2023.107838. 37295042

[18] A. M. Walling , N. L. Keating , K. L. Kahn , et al., “Lower Patient Ratings of Physician Communication Are Associated With Unmet Need for Symptom Management in Patients With Lung and Colorectal Cancer,” Journal of Oncology Practice 12, no. 6 (June 2016): e654–e669, 10.1200/JOP.2015.005538. PMC4957255 27221991

[19] P. Bolton , S. Dev , A. Giusto , et al., “Global Mental Health and Psychosocial Support Programming: An Expert Review of Major Implementation and Funding Challenges,” Global mental health (Cambridge, England) 12 (January 2025): e141, 10.1017/gmh.2025.10093. PMC12720214 41438270

[20] Marathon of Hope Cancer Centres Network . Patient Voices in Research Initiative. 2024. https://www.marathonofhopecancercentres.ca/funding-opportunities/program/patient-voices-in-research-initiative.

[21] L. C. Zwanenburg , M. L. van der Lee , J. J. Koldenhof , K. Suijkerbuijk , and M. Schellekens , “What Patients With Advanced Cancer Experience as Helpful in Navigating Their Life With a Long‐Term Response: A Qualitative Study,” Supportive care in cancer: official journal of the Multinational Association of Supportive Care in Cancer 32, no. 4 (March 2024): 222, 10.1007/s00520-024-08398-2. PMC10933145 38470541

[22] Marathon of Hope Cancer Centres Network . Patient Working Group 2024. https://www.marathonofhopecancercentres.ca/mohccn-working-groups/group/patient-working-group.

[23] S. Staniszewska , J. Brett , I. Simera , et al., “GRIPP2 Reporting Checklists: Tools to Improve Reporting of Patient and Public Involvement in Research,” BMJ 358 (August 2017): j3453, 10.1136/bmj.j3453. PMC5539518 28768629

[24] M. Q. Patton , Qualitative Research & Evaluation Methods. Fourth Edition (Sage, 2014).

[25] S. Scruton , C. Hovey , C. Kendell , and R. Urquhart , “Exploring the Experiences of Individuals Diagnosed With Metastatic Non‐Small‐Cell Lung Cancer: A Qualitative Study,” Current Oncology 32, no. 10 (October 2025): 570, 10.3390/curroncol32100570. PMC12564169 41149490

[26] B. Saunders , J. Sim , T. Kingstone , et al., “Saturation in Qualitative Research: Exploring Its Conceptualization and Operationalization,” Quality & quantity 52, no. 4 (2018): 1893–1907, 10.1007/s11135-017-0574-8. PMC5993836 29937585

[27] S. Hall and L. Liebenberg , “Qualitative Description as An Introductory Method to Qualitative Research for Master's‐Level Students and Research Trainees,” International Journal of Qualitative Methods 23 (May 2024): 16094069241242264, 10.1177/16094069241242264.

[28] M. Sandelowski , “What's in a Name? Qualitative Description Revisited,” Research in Nursing & Health 33, no. 1 (February 2010): 77–84, 10.1002/nur.20362. 20014004

[29] A. Tong , P. Sainsbury , and J. Craig , “Consolidated Criteria for Reporting Qualitative Research (Coreq): a 32‐item Checklist for Interviews and Focus Groups,” International Journal for Quality in Health Care 19, no. 6 (December 2007): 349–357, 10.1093/intqhc/mzm042. 17872937

[30] Canadian Cancer Society . 2023. Advanced Cancer, https://cancer.ca/en/cancer-information/resources/publications/advanced-cancer.

[31] D. Howell , D. Bryant Lukosius , J. Avery , et al., “A Web‐Based Cancer Self‐Management Program (I‐Can Manage) Targeting Treatment Toxicities and Health Behaviors: Human‐Centered Co‐Design Approach and Cognitive Think‐Aloud Usability Testing,” JMIR cancer 9 (July 2023): e44914, 10.2196/44914. PMC10403801 37477968

[32] S. Champ and C. Dixon , “Cancer Patient Navigation in Canada: Directions From the North,” Seminars in Oncology Nursing 40, no. 2 (April 2024;Patient Navigation): 151588, 10.1016/j.soncn.2024.151588. 38331627

[33] J. L. Bender , P. K. Flora , S. Soheilipour , et al., “Web‐Based Peer Navigation for Men With Prostate Cancer and Their Family Caregivers: A Pilot Feasibility Study,” Current Oncology 29, no. 6 (June 2022): 4285–4299, 10.3390/curroncol29060343. PMC9221974 35735452

[34] B. Pesut , W. Duggleby , G. Warner , et al., “Implementing Volunteer‐Navigation for Older Persons With Advanced Chronic Illness (Nav‐Care): a Knowledge to Action Study,” BMC Palliative care 19 (May 2020): 72, 10.1186/s12904-020-00578-1. PMC7245025 32443979

[35] R. J. Chan , V. E. Milch , F. Crawford‐Williams , et al., “Patient Navigation Across the Cancer Care Continuum: An Overview of Systematic Reviews and Emerging Literature,” CA: A Cancer Journal for Clinicians 73, no. 6 (2023): 565–589, 10.3322/caac.21788. 37358040

[36] Canadian Cancer Society . Connect With Our Online Community. N.d. https://cancer.ca/en/living-with-cancer/how-we-can-help/connect-with-our-online-community.

[37] Wellspring . Peer Support. Wellspring. N.d. https://wellspring.ca/program/peer-support/.

[38] M. Canada . Support groups for Myeloma patients. 2023. https://myeloma.ca/recently-diagnosed/find-support/.

[39] BC Cancer . Support Programs. N.d. https://www.bccancer.bc.ca:443/our-services/services/support-programs.

[40] A. Kiemen , M. Czornik , and J. Weis , “How Effective Is Peer‐To‐Peer Support in Cancer Patients and Survivors? A Systematic Review,” Journal of Cancer Research and Clinical Oncology 149, no. 11 (September 2023): 9461–9485, 10.1007/s00432-023-04753-8. PMC10374798 37120782

[41] L. Hemming , S. F. A. Duijts , N. Zomerdijk , et al., “A Systematic Review of Peer Support Interventions to Improve Psychosocial Functioning Among Cancer Survivors: Can Findings Be Translated to Survivors With a Rare Cancer Living Rurally?,” Orphanet Journal of Rare Diseases 19, no. 1 (December 2024): 473, 10.1186/s13023-024-03477-3. PMC11662424 39707418

[42] K. Hutchins , G. Hamlyn , A. Johnston , R. Thomas , J. Tian , and A. Kamal , “The Availability Versus Accessibility Gap in Supportive Care Services at 40 Nci‐Designated Cancer Centers,” Journal of Clinical Oncology 34, no. 7_suppl (March 2016): 283, 10.1200/jco.2016.34.7_suppl.283.

[43] D. Monnery , K. Law , D. P. Gopal , et al., “Models, Outcomes, Barriers, and Facilitators of Supportive Care in Cancer: A Scoping Review,” Supportive Care in Cancer: Official Journal of the Multinational Association of Supportive Care in Cancer 34, no. 4 (2026): 376, 10.1007/s00520-026-10613-1. PMC13031207 41893935

[44] J. Avery , H. K. Schulte , K. L. Campbell , et al., “What We Want Is More Access…”: Experiences of Supportive Cancer Care and Strategies for Advancement in a Canadian Provincial Cancer Care Organization,” Current Oncology 28, no. 3 (June 2021): 3, 10.3390/curroncol28030205. PMC8293065 34203638

[45] J. J. Sanders , S. Temin , A. Ghoshal , et al., “Palliative Care for Patients With Cancer: Asco Guideline Update,” Journal of Clinical Oncology 42, no. 19 (July 2024): 2336–2357, 10.1200/JCO.24.00542. 38748941

[46] A. Sarradon‐Eck , S. Besle , J. Troian , G. Capodano , and J. Mancini , “Understanding the Barriers to Introducing Early Palliative Care for Patients With Advanced Cancer: A Qualitative Study,” Journal of Palliative Medicine 22, no. 5 (May 2019): jpm.2018.0338, 10.1089/jpm.2018.0338. 30632886

[47] Canadian Virtual Hospice . Asked and Answered. N.d. https://www.virtualhospice.ca/en_US/Main+Site+Navigation/Home/Support/Support/Asked+and+Answered/Palliative+Care/How+is+eligibility+for+palliative+care+decided_+What+procedure+is+followed_.aspx.

[48] Windsor Regional Hospital . Palliative Care. N.d. https://www.wrh.on.ca/PalliativeCare.

[49] Winnipeg Regional Health Authority . Information for Health Care Professionals. N.d. https://wrha.mb.ca/palliative-care/information-for-health-care-professionals/.

[50] Canadian Partnership Against Cancer . Palliative care competency framework. N.d. https://www.partnershipagainstcancer.ca/topics/palliative-care-competency-framework/.

[51] Alberta Palliative Care Competency Frameworks . Services. N.d. https://www.albertahealthservices.ca/info/Page17398.aspx.

[52] Nova Scotia Health . The Nova Scotia Palliative Care Competency Framework: A Reference Guide for Health Professionals and Volunteers. 2017. https://library.nshealth.ca/PalliativeCare/Learn.

[53] S. E. Neil‐Sztramko , J. Smith‐Turchyn , A. Fong , K. Kauffeldt , and J. R. Tomasone , “Community‐Based Exercise Programs for Cancer Survivors: A Scoping Review of Program Characteristics Using the Consolidated Framework for Implementation Research,” Archives of Physical Medicine and Rehabilitation 103, no. 3 (March 2022): 542–558.e10, 10.1016/j.apmr.2021.06.026. 34375631

[54] A. Malakian , S. Mohammed , R. Fazelzad , R. Ajaj , A. Artemenko , and S. J. Mayo , “Nursing, Psychotherapy and Advanced Cancer: A Scoping Review,” European Journal of Oncology Nursing 56 (February 2022): 102090, 10.1016/j.ejon.2021.102090. 35026499

[55] G. Rodin , C. Lo , A. Rydall , et al., “Managing Cancer and Living Meaningfully (Calm): A Randomized Controlled Trial of a Psychological Intervention for Patients With Advanced Cancer,” Journal of Clinical Oncology 36, no. 23 (August 2018): 2422–2432, 10.1200/JCO.2017.77.1097. PMC6085180 29958037

[56] S. M. Shallwani , R. Thomas , S. Poitras , K. Toupin‐April , G. Sheill , and J. King , “Canadian Oncology Physiotherapists' Perspectives of Physical Activity in People With Advanced Cancer: A Mixed‐Methods Study,” Physiotherapy theory and practice 40, no. 6 (June 2024): 1281–1299, 10.1080/09593985.2022.2138663. 36305676

[57] G. Sheill , E. Guinan , L. O Neill , D. Hevey , and J. Hussey , “Physical Activity and Advanced Cancer: The Views of Chartered Physiotherapists in Ireland,” Physiotherapy theory and practice 34, no. 7 (July 2018): 534–541, 10.1080/09593985.2017.1422821. 29297722

[58] Y. Iwamitsu , A. Oba , K. Hirai , et al., “Troubles and Hardships Faced by Psychologists in Cancer Care,” Japanese Journal of Clinical Oncology 43, no. 3 (March 2013): 286–293, 10.1093/jjco/hys232. 23300170

[59] Canadian Cancer Research Alliance . Canada's Investment in Cancer Survivorship Research, 2005‐2019. 2019. https://www.ccra-acrc.ca/wp-content/uploads/2024/02/Surviv_2021_EN.pdf.

[60] R. Urquhart , L. Lethbridge , and G. A. Porter , “Patterns of Cancer Centre Follow‐Up Care for Survivors of Breast, Colorectal, Gynecologic, and Prostate Cancer,” Current Oncology 24, no. 6 (December 2017): 360–366, 10.3747/co.24.3627. PMC5736477 29270047

[61] O. Abdel‐Rahman , E. McFarlane , H. Ohm , et al., “Advances in Cancer Therapy Require Urgent Changes to the Oncology Workforce,” Canadian Medical Association Journal 197, no. 21 (June 2025): E590–E596, 10.1503/cmaj.241425. PMC12150415 40456575

[62] K. Jordan , M. Aapro , S. Kaasa , et al., “European Society for Medical Oncology (Esmo) Position Paper on Supportive and Palliative Care,” Annals of Oncology 29, no. 1 (January 2018 Focus on liquid bicopsy): 36–43, 10.1093/annonc/mdx757. 29253069

[63] L. C. Weeks , D. Seely , L. G. Balneaves , et al., “Canadian Integrative Oncology Research Priorities: Results of a Consensus‐Building Process,” Current Oncology 20, no. 4 (July 2013): 289–299, 10.3747/co.20.1378. PMC3728057 23904767

[64] Canadian Partnership Against Cancer . Models of Care Toolkit. https://www.partnershipagainstcancer.ca/topics/models-of-care/cancer-care-networks/multidisciplinary-clinics/.

[65] A. Chan , N. Nixon , M. Al‐Khaifi , et al., “Optimizing Adjuvant Care in Early Breast Cancer: Multidisciplinary Strategies and Innovative Models From Canadian Centers,” Current Oncology 32, no. 7 (July 2025): 402, 10.3390/curroncol32070402. PMC12293295 40710212

[66] C. J. L. Stone , A. P. Johnson , D. Robinson , et al., “Health Resource and Cost Savings Achieved in a Multidisciplinary Lung Cancer Clinic,” Current Oncology 28, no. 3 (April 2021): 1681–1695, 10.3390/curroncol28030157. PMC8161784 33947127

[67] M. Taberna , F. Gil Moncayo , E. Jané‐Salas , et al., “The Multidisciplinary Team (Mdt) Approach and Quality of Care,” Frontiers in Oncology 10 (March 2020): 85, 10.3389/fonc.2020.00085. PMC7100151 32266126

[68] A. Laupacis , “New Models of Care Needed to Address Canada's Shortage of Medical Specialists,” Canadian Medical Association Journal 197, no. 21 (June 2025): E597–E598, 10.1503/cmaj.250791. PMC12150409 40456572

[69] A. C. Tree , V. Harding , A. Bhangu , et al., “The Need for Multidisciplinarity in Specialist Training to Optimize Future Patient Care,” Nature Reviews Clinical Oncology 14, no. 8 (August 2017): 508–517, 10.1038/nrclinonc.2016.185. PMC7641875 27898067

[70] A. Reyes Veliz , J. Gray , and J. Karnon , “Economics of Multidisciplinary Teams in Oncology: A Scoping Review Protocol,” JBI evidence synthesis 18, no. 6 (June 2020): 1285–1291, 10.11124/JBISRIR-D-19-00103. 32813376

[71] Canadian Cancer Society . Health system funding. 2022. https://cancer.ca/en/get-involved/advocacy/what-we-are-doing/health-system-funding.

