# Supplementary material for: Identifying Patients' Priorities for Improving Supportive Care in Advanced Cancer: A Qualitative Study
Source: Health Expect. 2026 Nov 13;29(6):e70971. doi: 10.1111/hex.70971 (PMC13768803; doi:10.1111/hex.70971)
Supplement: Supplementary file 2 [file HEX-29-e70971-s002.pdf]

Codebook

Name	Sources	References
Current Healthcare Experiences	2	2
Description (being seen by, how often, reasons for being seen)	20	37
Diagnostic process	8	11
Issues with current care	19	103
Positives of current care	19	68
Demographics	21	28
Existing Supports	1	1
Healthcare system supports	19	79
Other supports	22	196
Future supportive care interventions	0	0
Components of interventions	20	74
Ideal healthcare system supports and processes	21	78
Information provision	17	40
Modality and design	7	8
Other considerations	17	36
Needs	1	1
Barriers to needs being met	22	213
Emotional Needs	22	109
Facilitators to needs being met	22	186
How supports are found or accessed	17	42
Physical Needs	22	136
Practical Needs	21	113
Unmet or Ongoing needs	5	6
