# Supplementary material for: Identifying Patients' Priorities for Improving Supportive Care in Advanced Cancer: A Qualitative Study
Source: Health Expect. 2026 Nov 13;29(6):e70971. doi: 10.1111/hex.70971 (PMC13768803; doi:10.1111/hex.70971)
Supplement: Supplementary file 4 [file HEX-29-e70971-s003.docx]

GRIPP2 - Short Form Checklist for the Reporting of Patient Engagement in Research

| **Section and topic** | **Item** | **Reported on lines** |
| --- | --- | --- |
| **1：Aim** | Report the aim of PPI in the study.  The goals of patient partner involvement in this study were to refine the research aims and questions, facilitate interviews to address the objectives of the study, and interpret the findings through the lens of lived experience. | Page 2, Paragraph 3 of Introduction. |
| **2 : Methods** | Provide a clear description of the methods used for PPI in the study  ***Patient and Public Involvement***  Three patient partners were actively and meaningfully engaged as a part of the research team throughout the entire study process. Their involvement across all stages of the study is detailed throughout the Methods section. These individuals were living with an advanced cancer diagnosis and could apply their lived experience to the design and interpretation of the study. The patient partners included two working-age women living in urban settings, and one retired man living in a rural setting.  In addition, this study was designed to address the research goals established by the MOHCCN Patient Working Group (PWG) as a part of the Patient Voices in Research Initiative. The PWG is a group of over 30 volunteers with lived cancer experience who work closely with researchers and physicians to shape cancer care in Canada. The group represents diverse ages, genders, ethnicities, educational backgrounds, cancer sites, and geographic regions across Canada. The PWG helped to guide the study development and interpretation of findings through regular updates and meetings with the research team.  Patient and public involvement in this study is reported using the Guidance for Reporting Involvement of Patients and the Public (GRIPP2) shortform checklist (Appendix 1). | Page 3-5, Paragraphs 2-4, and 7 of Methods |
| **3：Study Results** | Outcomes—Report the results of PPI in the study, including both positive and negative outcomes  Patient partners contributed to the development and refinement of the interview guide and participated in the analysis of the data, as described in the Methods. Their perspectives informed the interpretation of participants’ accounts and contributed to the development of the final themes and findings reported below. No negative outcomes or challenges arising from patient and public involvement were identified. | Page 5, Paragraph 2 of Results |
| **4：Discussion and Conclusions** | Outcomes—Comment on the extent to which PPI influenced the study overall. Describe positive and negative effects  The involvement of patient partners and the MOHCCN PWG influenced the study by bringing patient perspectives into the design of the study and interpretation of the findings. Their involvement provided an additional perspective alongside those of researchers and clinicians to ensure the interpretation of participants’ experiences remained grounded in patient perspectives. We did not identify any negative effects of PPI. | Page 14, Paragraph 7 of Discussion |
| **5：Reflections/Critical Perspective** | Comment critically on the study, reflecting on the things that went well and those that did not, so others can learn from this experience  This study is not without its limitations. The self-selected sample had limited representation of men, individuals without post-secondary education, and individuals living in rural or remote areas. As such, the results cannot be generalized to all individuals who are diagnosed with advanced cancer and may not reflect the experiences of those who face additional barriers to care. However, we achieved theoretical saturation with a relatively large sample size, which is a key strength that supports the credibility and robustness of the findings and allows us to demonstrate patient priorities for improving cancer care. In addition, race and ethnicity were not collected, limiting our ability to assess how these factors may have shaped participants’ experiences and priorities. | Page 15, Paragraph 1 of Limitations |

PPI: Patient and Public Involvement

*Staniszewska S, et al. GRIPP2 reporting checklists: tools to improve reporting of patient and public involvement in research. Research Involvement and Engagement. 2017;3:13.*
